# Investigation of Multilayer Nanostructures of Magnetic Straintronics Based on the Anisotropic Magnetoresistive Effect

**DOI:** 10.3390/s21175785

**Published:** 2021-08-27

**Authors:** Dmitry Zhukov, Vladimir Amelichev, Sergey Kasatkin, Dmitry Kostyuk

**Affiliations:** Scientific-Manufacturing Complex “Technological Centre”, 124498 Moscow, Russia; V.Amelichev@tcen.ru (V.A.); kasatkin14@mail.ru (S.K.); D.Kostyuk@tcen.ru (D.K.)

**Keywords:** magnetostriction effect, magnetoresistive effect, thin-film nanostructures, magnetic straintronics

## Abstract

The article presents the results of experimental studies of multilayer nanostructures of magnetic straintronics formed by magnetron sputtering on a 100 mm silicon wafer. The object of the study is two types of nanostructures: Ta/FeNiCo/CoFe/Ta and Ta/FeNi/CoFe/Ta, differing in the ratio of magnetic layers. The magnetic and magnetoresistive characteristics of multilayer nanostructures under varying mechanical loads are studied both on a 100 mm wafer and in the form of 4 × 20 mm^2^ samples of two types. The first, where the axis of easy magnetization is directed along the long side of the sample, and the second, where the axis of easy magnetization is a tilt at 45°. Based on the obtained data, the conclusions about the practical application of these nanostructures in magnetic straintronics elements are drawn.

## 1. Introduction

Straintronics is a branch of nanoelectronics in which the connection between different physical phenomena is carried out by means of mechanical deformation of a thin-film nanostructure. The deformation in the layers of a thin-film nanostructure under the influence of external control fields makes changes in the electrical, magnetic and mechanical parameters of materials. It makes it possible to implement a fundamentally new generation of microelectronic devices and devices that combine various physical effects. Magnetic straintronics is based on the effects of direct and reverse magnetostriction in thin films made of different materials and alloys that are included in the composition of multilayer nanostructures.

The market for combined sensors, transducers and MEMS is predicted by Marketwatch to grow by an average of 11.74% by 2025. This forecast is based on an increase in global demand for portable and wearable devices; in addition, demand for combined microelectronic devices will increase in the consumer markets: electronics, Internet of smart things (IoT), automotive, healthcare, aerospace and defense industries [1,2,3,4,5]. According to estimates, the market for magnetosensitive transducers by 2027 will reach a value of USD ~4.7 billion, with an increase over the forecast period by an average of 6% [6].

In the elements of magnetic straintronics based on thin-film nanostructures, the acting mechanical stresses lead to a change in the magnetic properties of these nanostructures. Therefore, when nanostructures contain a complex of connected magnetostrictive and magnetoresistive layers, it is possible to establish a relationship between induced mechanical deformations and the output electrical signal.

If this film is bonded in a nanostructure with another magnetic film with magnetostrictive properties, then the device becomes sensitive to mechanical deformations, which expands the functional scope of such materials for a number of devices and devices. Of interest is the approach, the essence of which is the spatial separation of the ferromagnetic medium into two layers: one of which provides a high value of the magnetoresistive effect, and the other provides a high value of the magnetostrictive effect [7]. A necessary requirement, in this case, is effective exchange interaction between the layers. In such a multilayer ferromagnetic nanostructure, upon its mechanical deformation, the magnetic state of the magnetostrictive layer changes, which entails a change in the magnetic state of the magnetoresistive layer. The magnitude of the magnetoresistive effect in a multilayer nanostructure can depend on both the layer thicknesses and the ratio of the layer thicknesses. In addition, it is possible that structural conformity at the interface between layers may affect mechanical stresses. Thus, in this nanostructure, it is possible to combine the effects of spintronics and straintronics.

Therefore, there is a special interest in studying nanostructures containing magnetoresistive and magnetostrictive materials in a certain ratio. Thin FeNiCo layers have a high anisotropic magnetoresistive (AMR) effect (~2–4%) and a low magnetostriction coefficient [8,9,10]. In the Co_50_Fe_50_ layers, a low AMR effect (~0.3%) and a significant magnetostrictive effect are observed [8,9,10,11,12,13,14].

At present, an alloy based on Fe–Co is a promising material for straintronics. For example, in layers of the composition Fe_66_Co_34_ after appropriate heat treatment, the magnetostriction coefficient reached a value of ~250 ppm [12]. However, the nature of the proposed heat treatment is incompatible with the technological routes of manufacturing some devices; in addition, the layers have a high coercivity value Hc ≥ 4 kA/m.

The magnetostrictive properties of Fe–Co alloy films are manifested in a wide range of the composition of this alloy, which allows us to hope for the optimization of such a parameter as the magnetic anisotropy field while maintaining the magnetostrictive properties. Another possibility of reducing the magnitude of the magnetic anisotropy field is associated with the use of sublayers that actively affect the orientation of the crystal texture during the deposition of Fe–Co layers [13]. It is known that values of the magnetostriction coefficient up to 60 ppm are achievable under heat treatment modes up to 400 °C.

The results of [12,13,14] theoretical studies of the Co–Fe phase diagram are known, and experimental confirmation of the possibility of realizing high values of the magnetostriction coefficients for compositions 0.5 < x < 0.7 is presented, along with the possibility of obtaining layers of such alloys with a magnetostriction coefficient of up to 150 ppm with slow cooling and moderate annealing temperatures. Thus, it is obvious that the use of Fe–Co layers for the manufacture of magnetic straintronics devices is relevant and requires quite definite research and technological efforts.

In nanostructures based on the giant magnetoresistive effect (GMR), which has a magnetostrictive layer upon mechanical deformation due to the effect of reverse magnetostriction (Villari effect), the magnetic state of the free layer changes, which in turn leads to a change in the magnetoresistive effect of the nanostructure. In this case, the free layer of the GMR nanostructure should have magnetostrictive properties.

In [15], the results of experimental studies of multilayer nanostructures based on the GMR effect, containing magnetostrictive FeCo layers as a free layer, are presented. NiFe/Cu/Co/FeMn, FeCo/Cu/Co and FeCo/Cu/FeCo/FeMn nanostructures were investigated, the magnetoresistive effect was 3.4%.

In [16], the results of experimental studies of the GMR of the Ta/FeNiCo/CoFe/Cu/CoFe/FeNiCo/FeMn/Ta nanostructure formed on an oxidized silicon substrate were presented. The effect of mechanical deformation on the electrophysical parameters of the nanostructure was investigated.

Of greatest interest is a nanostructure with a magnetic tunnel junction on MgO and the effect of mechanical stress on its properties [2]. In [17], the results of theoretical and experimental studies of the tunnel junction with CoFeB/MgO/CoFeB were presented. It was confirmed that the mechanical stress of stretching the tunnel junction changes its magnetic properties.

A nanostructure with a tunneling magnetic junction containing a magnetostrictive and magnetoresistive layer and formed on a membrane was described in [18]. The article described the PtMn/CoFeB/Ru/CoFeB nanostructure and magnetostrictive FeCoSiB layer with a dielectric MgO layer. The sensitivity of the nanostructure to mechanical stress is 840. Article [19] described a tunnel junction with a magnetostrictive CoFe layer and a dielectric Al_2_O_3_ layer; the magnitude of the magnetoresistive effect is 48%. The sensitivity to mechanical stress of the nanostructure is at least 600.

The gauge factor (GF) of a transducer is equal to the relative change in resistance divided by the strain. The metallic-based strain sensors are characterized by the value GF = 2–5, and the piezoelectric based on silicon GF = 100–140 [18,20].

To study the properties of multilayer periodic nanostructures based on magnetoresistive nanolayers Fe_16_Ni_64_Co_20_ or Fe_19_Ni_81_ and magnetostrictive CoFe, multilayer nanostructures were developed (Table 1). These nanostructures are manufactured at the Scientific-Manufacturing Complex “Technological centre”.

The main goal is to search for the optimal structure for magnetic straintronics elements based on the magnetoresistive and magnetostrictive effects. Fundamental scientific studies of these periodic nanostructures and their potential application in magnetic field-conducting nanoelectronics are also very important.

## 2. Measurement Technology and Methods

The nanostructures formed by magnetron sputtering, where oxidized silicon wafers with a diameter of 100 mm and a thickness of 0.46 mm, were used as the initial substrates. The axis of easy magnetization (EMA) in nanostructures formed with sputtering under the influence of a constant magnetic field of ~100 Oe in the plane of the substrate. Ta layers with a thickness of 5 nm were used as a buffer and protective coating.

**Figure 1:**
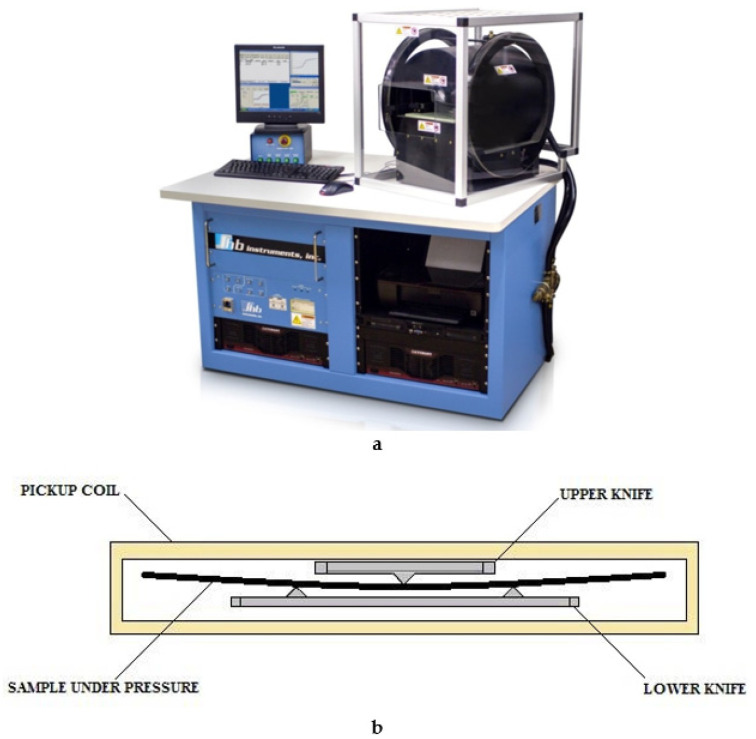
(**a**) Equipment MESA-200. (**b**) Schematic representation of a block for creating mechanical stresses on a plate on the MESA-200 equipment.

The used control and measuring equipment included a MESA-200 measuring installation from Shb Instruments (USA) with software for control, programming, remote control and diagnostics, as well as a personal computer with installed software [9,16]. MESA-200 equipment (Figure 1a) allows measuring the magnetic parameters of magnetoresistive structures, as part of silicon wafers up to 200 mm in diameter, in a constant and alternating magnetic field up to 80 kA/m, as well as an inductance field range of 0.01–10,000 nVb/div. The main features are the measurement of the magnetoresistive effect, measurements with a frequency of changes in the magnetic field up to 10 Hz (10 loops per second), digital filtering of background noise to suppress the influence of external fields and suppress the influence of the earth’s magnetic field. To create the required magnetic field, the measuring system includes a 400 mm main axis solenoid and a 450 mm transverse solenoid. The maximum magnetizing field is 80 kA/m along the main axis and 8 kA/m along the transverse axis.

The formed nanostructures on silicon substrates were used to study the magnetic parameters both in the state of mechanical compression deformation and without any on a specialized MESA-200 measuring unit (a schematic representation of the main unit of equipment is shown in Figure 1b). Controlled mechanical stress in the unit led to deformation (compression) of the nanostructure on the silicon substrate. The direction of these stresses is perpendicular to the magnetic field created by the unit.

**Figure 2:**
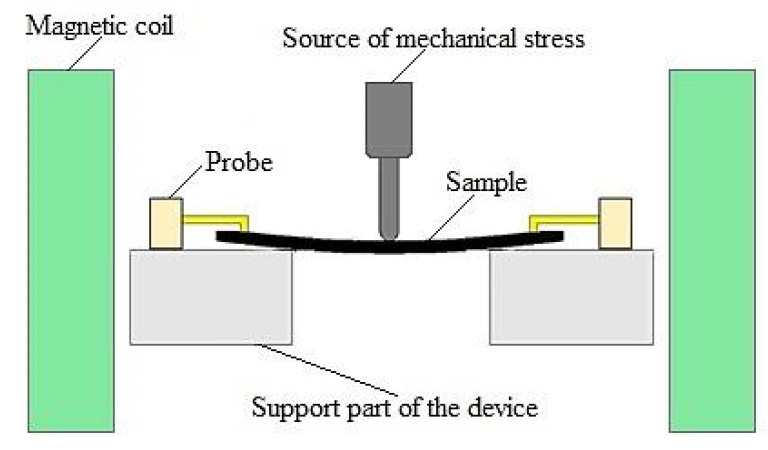
Schematic representation of the main setup unit.

After studying the nanostructures on the wafer, two types of samples 4 × 20 mm^2^ were studied: in the first, the EMA was directed along the long side of the sample; in the second, the EMA was tilted at 45° to the long side and measuring the magnitude of the AMR effect under conditions of varying mechanical load. The AMR effect was measured by a two-probe method—the unit has a device for creating controlled mechanical deformations in the surface layer of the sample [8,16,21]. The sketch of the main unit of the installation is shown in Figure 2.

The installation is intended for studying nanostructures of magnetic straintronics, which have a magnetoresistive effect. The mechanical part of the device includes a device for creating mechanical pressure on the sample and two Helmholtz coils that create a magnetic field. The sample is pressurized with a 7.94 μm linear stepper motor. The force of pressure is measured by a compression–tension strain gauge. The connection of the measuring setup with a personal computer is provided by means of a USB cable. The device is controlled, the measurement parameters are set (such as the magnitude of the mechanical stress on the sample, the magnitude of the magnetic field, etc.), and the storage of the results is carried out from a computer using a developed specialized program. The installation allows for studies of nanostructures and provides a magnetic field up to 300 Oe and maximum pressure on the sample—10 N. In the installation, it is possible to create both compression deformation of the sample and tension.

## 3. Results and Discussion

According to the results of the study of nanostructures on a specialized MESA-200 unit, the dependences of the magnetization reversal *B*(*H*) for each type of nanostructures were obtained. These characteristics lead to a general conclusion that magnetic anisotropy is observed in the initial state of the nanostructures. When applying a compression load to the sample along the EMA, the shape of the magnetization reversal curve changes and the coercivity of the nanostructure increases, but the application of compressive stresses perpendicular to the EMA does not significantly change the shape of the magnetization reversal loop.

**Figure 3:**
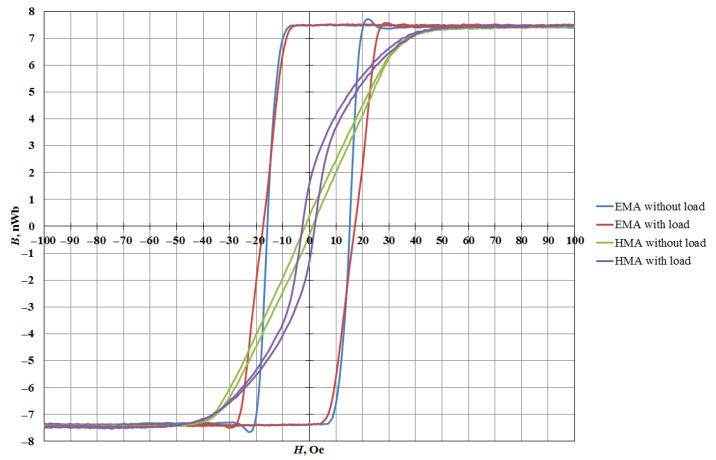
Dependence of *B*(*H*) in nanostructure Ta (5 nm)/FeNiCo (30 nm)/CoFe (10 nm)/Ta (5 nm) with and without mechanical load.

This relationship is demonstrated in Figure 3—*B*(*H*) characteristic of the Ta (5 nm)/FeNiCo (30 nm)/CoFe (10 nm)/Ta (5 nm) nanostructure. The figure clearly shows that when a compression load is applied to the sample along the EMA, the coercivity of the nanostructure changes from 1.3 Oe to 2.4 Oe. The change in the loop of the axis of hard magnetization (HMA) and its partial reversal to the EMA can be explained by the appearance of magnetic anisotropy induced by mechanical stress [2]. During the compression load along the HMA, the coercivity of the nanostructure increased from 15.8 Oe to 17.1 Oe, and the loop did not change.

Next, all seven types of nanostructures were studied on 4 × 20 mm^2^ samples of two types. The first had the EMA directed along the long side of the sample. The second sample had the EMA at a 45° tilt to the long side. The dependences of the AMR effect are on the magnitude of the magnetic field under mechanical load. The parameters were obtained for all types of samples, and the article presents the dependencies for the sample, Ta (5 nm)/FeNiCo (30 nm)/CoFe (10 nm)/Ta (5 nm) as an example.

**Figure 4:**
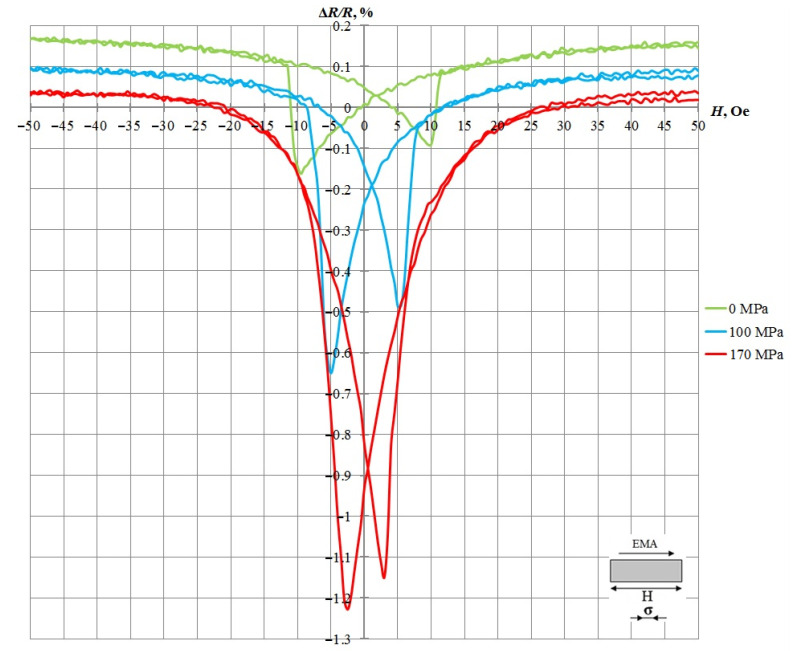
Results of measurement of the AMR effect with and without mechanical load.

Figure 4 shows the dependence of the AMR effect of a nanostructure in the state of free and deformed compression (170 MPa) on the magnitude of the external magnetic field in a sample with EMA formed along the long side.

For the studied sample in the absence of mechanical load, the AMR effect of 0.28 was determined at a coercive force of 9.5 Oe. Under the influence of mechanical compression deformation of 170 MPa, the AMR effect increased to 1.22, and the value of the coercive force was 2.6 Oe. Thus, the relative change in resistance due to the load (∆*R/R*)_σ_ is 0.94.

**Figure 5:**
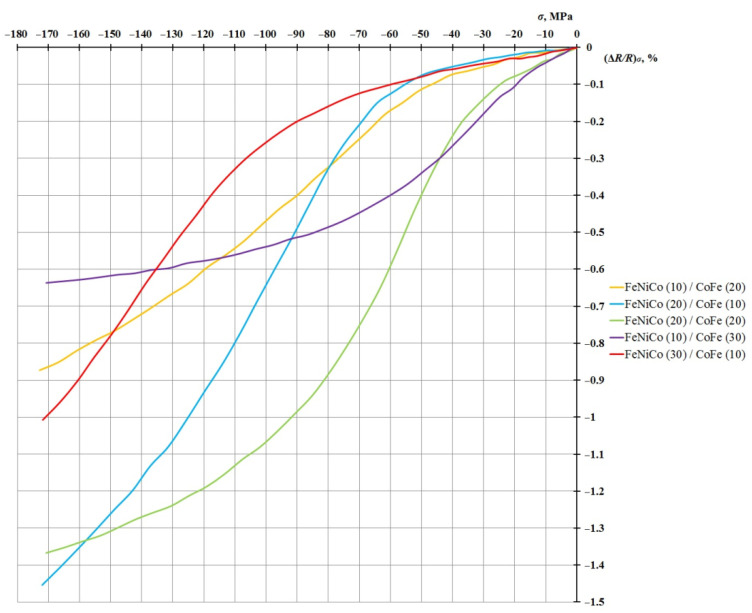
Dependence of the value of the relative change in the resistance due to the load (Δ*R/R*)*_σ_* on the value of mechanical load *σ* for nanostructures of the FeNiCo/CoFe type (layer thickness in nm).

Figure 5 shows the dependence of the magnetoresistance due to the mechanical load (∆*R/R*)*_σ_* on the value of the mechanical compression stress for five samples of FeNiCo/CoFe-type nanostructure, with EMA along the long side.

This graph shows that the FeNiCo (10 nm)/CoFe (30 nm) structure has a higher sensitivity in the region of small deformations transiting to saturation. The other four nanostructures of this type are characterized by low susceptibility at low-stress values and a sharp increase in the AMR effect with an increase in the mechanical compression of the nanostructure (more than 40 MPa). This effect can be explained by a model of the exchange–elastic interaction at the interface of ferromagnetic layers with different coercivities [22,23,24]. Thus, nanostructures exhibit different degrees of susceptibility to mechanical compression deformation. This is a prerequisite for using them in various sensitive elements of microelectronics, depending on the requirements. The particular interest is the analysis of the output characteristics of the sample pairs:

₋ FeNiCo (20 nm)/CoFe (10 nm) and FeNi (20 nm)/CoFe (10 nm);
₋ FeNiCo (10 nm)/CoFe (20 nm) and FeNi (10 nm)/CoFe (20 nm).

**Figure 6:**
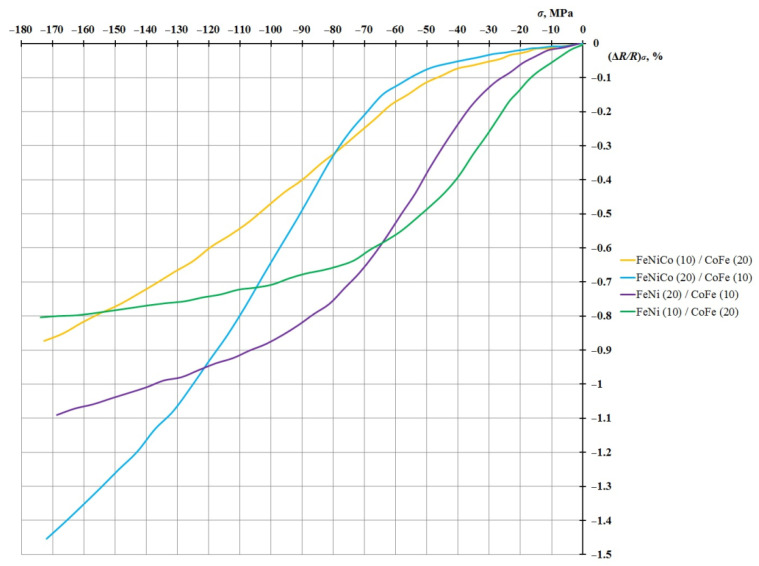
Dependence of the relative change in the resistance due to the load (Δ*R/R*)*_σ_* on the value of mechanical stresses *σ* for the FeNiCo/CoFe and FeNi/CoFe nanostructures (layer thickness in nm).

The dependence of the value of the relative change in the resistance due to the load (Δ*R/R*)*_σ_* on the value of mechanical stresses *σ* for these four nanostructures is shown in Figure 6.

This graph shows that although FeNi/CoFe nanostructures do not exceed similar FeNiCo/CoFe nanostructures in the AMR effect, they are characterized by a higher sensitivity to small deformations.

The sensitivity of magnetoresistive elements to mechanical deformation can be estimated as *GF* = *(*∆*R/R)_σ_*/Δ*ε*, where Δ*ε* is the deformation of the sensitive layer due to mechanical load within the elastic limits, $\Delta \varepsilon =\frac{\Delta \sigma }{E_{f}}$, where *E_f_* is Young’s modulus for the structure (110 GPa). The results of measuring relative change in resistance due to the load and the sensitivity coefficient for the seven studied nanostructures under mechanical compression deformation of 170 MPa are presented in Table 2.

**Figure 7:**
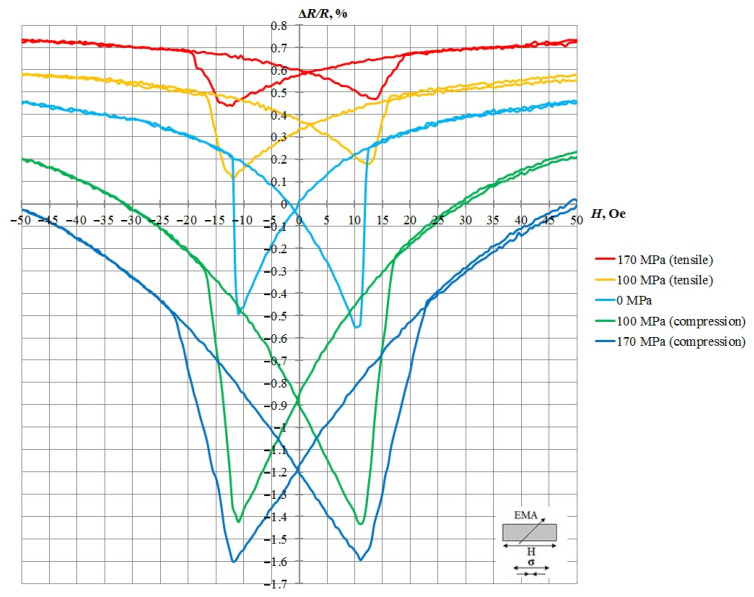
Results of measuring the magnetoresistive effect in the absence and presence of mechanical compression/tension load.

Figure 7 shows the dependence of the AMR effect for a sample where the EMA is tilted at 45° to the long side of the sample. The magnetic characteristics of the nanostructures were measured at mechanical compressive/tensile stresses of ±170 MPa. Higher voltages lead to the risk of the silicon substrate’s destruction. The ratio of the axes is shown in Figure 7.

A magnetoresistive effect of 1.0 with a coercivity of 10.5 Oe registered in the sample without applying mechanical deformation. Under the influence of mechanical compression deformation of 170 MPa, the magnetoresistive effect of the nanostructure increased to 1.59, and the coercivity increased to 11.4 Oe. When a mechanical extension stress of 170 MPa was applied to the sample, the magnetoresistive effect decreased to 0.27, and the coercivity increased to 12.9 Oe. Therefore, the relative change in resistance due to the load (∆*R/R*)_σ_ reached 1.32. The resulting graph allows us to conclude that the nanostructure reacts to both compression and tension.

**Figure 8:**
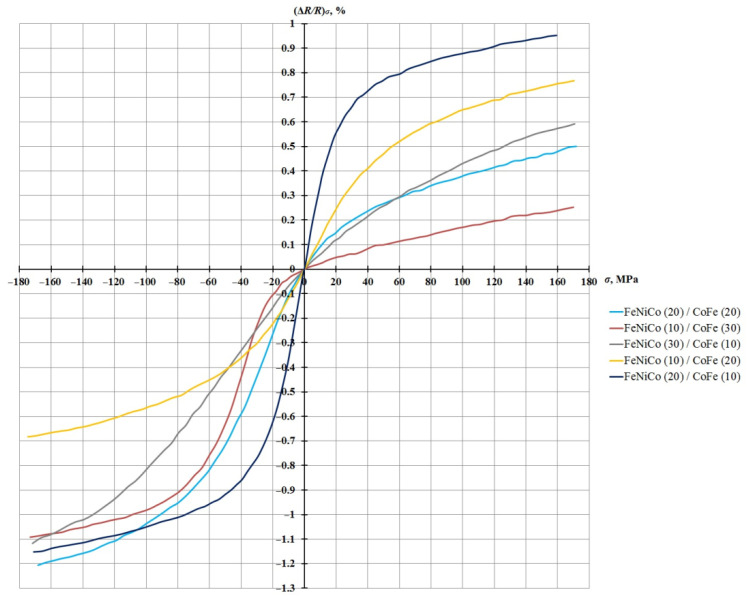
Graph of the dependence of the relative change in resistance due to the load on the mechanical stresses of compression/extension (layer thickness in nm).

The change in the resistance due to mechanical load (∆*R/R*)*_σ_* depending on the value of the mechanical compression and tension stress ±170 MPa, for five FeNiCo/CoFe-type nanostructures, with EMA at an angle of 45° to the long side of the sample, is shown in Figure 8.

Figure 8 shows that the FeNiCo (20 nm)/CoFe (10 nm) nanostructure has a higher sensitivity in the region of small deformations. All the studied nanostructures react to mechanical compression and tension deformation, but the FeNiCo (10 nm)/CoFe (30 nm) and FeNiCo (20 nm)/CoFe (20 nm) nanostructures show low sensitivity to tensile deformation, which is probably explained by the increased content of CoFe, characterized by higher coercivity compared to FeNiCo, in these nanostructures.

Thus, nanostructures exhibit different degrees of susceptibility to mechanical deformation of compression and tension and are able to find applications in sensitive microelectronics elements, depending on the requirements.

**Figure 9:**
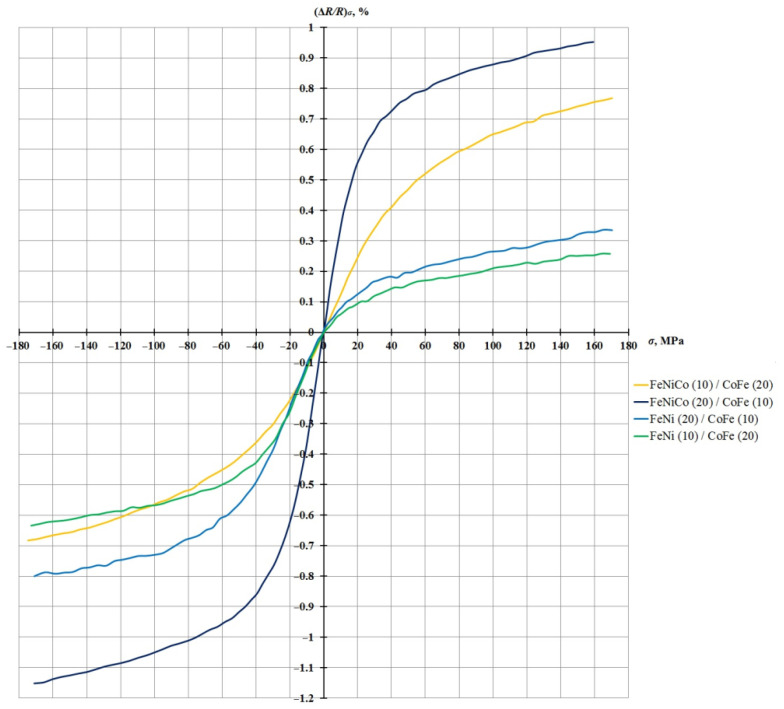
Graph of the dependence of the value of the relative change in the resistance due to the load on the value of the mechanical compressive/tensile stresses for the FeNiCo/CoFe and FeNi/CoFe nanostructures (layer thickness in nm).

Figure 9 shows the output characteristics of the image pairs FeNiCo (20 nm)/CoFe (10 nm) with FeNi (20 nm)/CoFe (10 nm) and FeNiCo (10 nm)/CoFe (20 nm) with FeNi (10 nm)/CoFe (20 nm).

Figure 9 shows that FeNi/CoFe nanostructures have a small value of the relative change in resistance due to the load and do not exceed similar FeNiCo/CoFe nanostructures in sensitivity.

The results of calculating the sensitivity of magnetoresistive elements to mechanical compression/tension stresses and the total value of the relative change in resistance due to the load for the seven investigated nanostructures on the linear section of mechanical stress are presented in Table 3.

The authors believe that changes in the magnetoresistance of multilayer nanostructures under the action of a magnetic field at a constant value of mechanical stress are determined, first of all, by deviations of the magnetization vectors of ferromagnetic films. A similar model for spin-tunneling magnetoresistive nanostructures based on the theory of micromagnetism was presented in [1] and, according to the authors, explains the dependence of the change in the magnetoresistance of nanostructures on the action of a magnetic field for a simpler case of anisotropic nanostructures. The change in magnetic anisotropy occurs mainly when the acting mechanical stress on nanostructures changes and affects the magnitude and type of change in magnetoresistance under the action of a magnetic field, which is confirmed by the measurement results.

## 4. Conclusions

As a result, nanostructures containing a magnetostrictive layer of CoFe and a magnetoresistive layer of FeNiCo or FeNi were manufactured and studied. The study of the samples showed that the nanostructures react to both compression and tension deformations. Applying mechanical compression stress to the nanostructure leads to a change in the AMR affect up to 1.46, and in the case of compression/tension deformation, the total value of the relative change in resistance due to the load reaches 2.1. The Ta (5 nm)/FeNiCo (20 nm)/CoFe (10 nm)/Ta (5 nm) nanostructure shows the largest sensitivity to mechanical stresses, which may be explained by a more optimal ratio of magnetic layers in the structure.

The comparison of similar nanostructures in terms of ratio, but different from each other in the FeNiCo or FeNi material, showed that nanostructures with FeNiCo are superior to nanostructures with FeNi as a magnetoresistive material in terms of the AMR effect and sensitivity.

The experimental studies confirmed the possibility of using multilayer nanostructures as a sensitive element in magnetic straintronics devices, where both mechanical compression and tension deformation can act as an external influencing factor. It significantly expands the functional range of such nanostructures in strain transformation devices based on magnetic straintronics.

## Figures and Tables

**Table 1:** Parameters of the studied nanostructures.

№	Nanostructure	Layer Thickness, nm				
		Structure 1	Structure 2	Structure 3	Structure 4	Structure 5
1	Ta	5	5	5	5	5
	FeNiCo	10	20	20	30	10
	CoFe	20	10	20	10	30
	Ta	5	5	5	5	5
2	Ta	5	5			
	FeNi	10	20			
	CoFe	20	10			
	Ta	5	5			

**Table 2:** Magnetoresistive characteristics of nanostructures (layer thickness in nm).

	Ta (5)	Ta (5)	Ta (5)	Ta (5)	Ta (5)	Ta (5)	Ta (5)
	FeNiCo (20)	FeNiCo (10)	FeNiCo (30)	FeNiCo (20)	FeNiCo (10)	FeNi (20)	FeNi (10)
	CoFe (20)	CoFe (30)	CoFe (10)	CoFe (10)	CoFe (20)	CoFe (10)	CoFe (20)
	Ta (5)	Ta (5)	Ta (5)	Ta (5)	Ta (5)	Ta (5)	Ta (5)
(Δ*R/R*)*_σ_*, %	1.36	0.64	1.05	1.46	0.88	1.10	0.81
*GF*	19.9	8.8	13.0	16.8	8.2	15.5	13.8

**Table 3:** Sensitivity of nanostructures to mechanical compressive/tensile stresses (layer thickness in nm).

	Ta (5)	Ta (5)	Ta (5)	Ta (5)	Ta (5)	Ta (5)	Ta (5)
	FeNiCo (20)	FeNiCo (10)	FeNiCo (30)	FeNiCo (20)	FeNiCo (10)	FeNi (20)	FeNi (10)
	CoFe (20)	CoFe (30)	CoFe (10)	CoFe (10)	CoFe (20)	CoFe (10)	CoFe (20)
	Ta (5)	Ta (5)	Ta (5)	Ta (5)	Ta (5)	Ta (5)	Ta (5)
|(Δ*R/R*)*_σ_*|, %	1.7	1.3	1.7	2.1	1.5	1.1	0.9
*GF*	11.1	6.4	7.3	22.2	10.4	9.4	7.9

## Data Availability

Not applicable.
